# A standardized educational implementation strategy improves operational Code Sepsis knowledge in a high-complexity hospital in Colombia

**DOI:** 10.3389/frhs.2026.1907012

**Published:** 2026-08-04

**Authors:** Laura M. Castillo-Morales, Camilo Vega-Useche, Germán Devia Jaramillo

**Affiliations:** 1 Department of Critical Medicine and Intensive Care, Fundación Santa Fe de Bogotá, Bogotá, Colombia; 2 Universidad Pedagógica y Tecnológica de Colombia, Tunja, Colombia; 3 School of Medicine, Universidad del Rosario, Bogotá, Colombia; 4 Emergency Department, Fundación Santa Fe de Bogotá, Bogotá, Colombia

**Keywords:** Code Sepsis, continuing education, educational intervention, hospital personnel, implementation science, quality improvement, sepsis

## Abstract

**Background:**

Timely recognition and early bundle-based care are essential to improve sepsis outcomes. However, implementation gaps persist in routine hospital practice, particularly in middle-income settings where multidisciplinary coordination and operational knowledge of institutional protocols are critical. This study evaluated the effect of a standardized educational strategy on immediate Code Sepsis knowledge among hospital personnel in a high-complexity hospital in Colombia.

**Methods:**

We conducted a single-group quasi-experimental before-and-after study in 2025 among clinical and non-clinical hospital personnel who voluntarily attended a 45-minute in-person multidisciplinary training session on the institutional Code Sepsis strategy. Participants completed a 12-item expert-reviewed assessment before and after intervention. Because one implementation-status item presented a postintervention response-format artifact, the primary outcome was an 11-item knowledge score ranging from 0 to 11. Strict linkage yielded 591 complete pre–post pairs. Paired score changes were evaluated using bootstrap confidence intervals, the Wilcoxon signed-rank test, and rank-biserial correlation. Item-level changes were assessed using McNemar's test with false discovery rate adjustment, while generalized estimating equations were used to estimate the overall postintervention change in the odds of a correct response.

**Results:**

The mean 11-item score increased from 5.80 to 9.22. The paired mean increase was 3.42 points (95% CI, 3.20–3.64), representing 31.1% of the total possible score range. The median paired increase was 3.0 points, and the rank-biserial correlation was 0.9646, indicating a very large favorable paired effect. Overall, 88.0% of participants improved, whereas 5.4% obtained a lower postintervention score. All 11 items improved after false discovery rate correction. Correct item-level responses increased from 52.7% to 83.8%, and postintervention assessments were associated with 5.63-fold higher odds of a correct response (95% CI, 5.05–6.28; *p* < 0.001). Sensitivity analyses yielded closely similar estimates, supporting the consistency of the observed improvement.

**Conclusions:**

A brief, standardized, multidisciplinary educational strategy was associated with a substantial and consistently favorable immediate improvement in operational Code Sepsis knowledge among hospital personnel. These findings reflect immediate knowledge acquisition and should not be extrapolated to long-term retention, changes in clinical practice, or patient outcomes.

## Introduction

Sepsis is defined as life-threatening organ dysfunction caused by a dysregulated host response to infection (1). It is a worldwide health concern, with 48.9 million new cases and 11 million associated deaths globally (2). In developed countries, it has been estimated that up to one-third or more of in-hospital deaths occur in patients with sepsis (3).

In Colombia, sepsis, particularly septic shock, is associated with a high mortality rate. In a multicenter, prospective cohort study conducted at 10 university hospitals in major cities across the country, the 28-day mortality rate for septic shock was 45.6% (4). Furthermore, at a high-complexity hospital in Bogotá, the mortality from septic shock ranged from 56.4% to 61.3% in patients treated in emergency rooms (5).

In recognition of this threat, the World Health Organization adopted a resolution in 2017 declaring sepsis a global health priority and urged countries to improve its prevention, diagnosis, and timely management (6). Early management of sepsis is crucial to improve the prognosis. International guidelines, led by the Surviving Sepsis campaign, emphasize the importance of promptly identifying septic patients and initiating resuscitative measures (7). The 2026 Surviving Sepsis Campaign pediatric guidelines similarly reinforce the need for timely recognition, structured reassessment, age-appropriate hemodynamic support, antimicrobial therapy, and source control in children with sepsis or septic shock (8). Because these recommendations are population-specific, they complement rather than replace the adult guidance underpinning the institutional protocol evaluated in this study.

In practice, this involves implementing a comprehensive set of essential interventions within a specific timeframe. These interventions include early administration of broad-spectrum antibiotics, prompt collection of blood cultures, measurement of serum lactate levels, resuscitation with intravenous fluids, vasopressor support to maintain appropriate mean arterial pressure, and rapid control of the infectious source (7, 9). Following these measures is associated with increased survival (10–12), and a lower mortality rate has been described when appropriate antibiotics are administered within the first hour of recognizing sepsis (13). To standardize and expedite patient care, several institutions have implemented sepsis protocols. These protocols are activated upon suspicion of sepsis or septic shock to facilitate an early multidisciplinary response (14, 15). Beyond protocol availability, recent developments emphasize the potential value of connected and interoperable critical care data infrastructures for benchmarking, education, quality improvement, and real-world clinical decision support in sepsis. However, their routine implementation remains constrained by challenges related to data harmonization, interoperability, governance, and privacy (16).

Quasi-experimental studies have documented enhancements in bundle compliance and a decrease in mortality rates following its implementation, both in Europe and Colombia (12, 17). However, a discrepancy persists between established guidelines and actual practice, attributed to systemic and human obstacles, including deficiencies in knowledge, situational awareness, and staff training necessary for the prompt recognition and management of sepsis (13, 18). Knowledge gaps and limited operational familiarity with sepsis recognition and time-sensitive care processes have been consistently documented among healthcare professionals (19–21). Therefore, institutional sepsis strategies require not only formal protocols, but also deliberate efforts to strengthen workforce preparedness for early recognition and coordinated response.

In this context, educational strategies constitute a fundamental element for enhancing protocol knowledge, practical recognition of time-sensitive actions, and the implementation of institution-specific sepsis workflows. Reviews and multicenter programs indicate that training correlates with improvements in care processes and, in certain instances, with enhanced clinical outcomes, particularly when incorporated into institutional quality improvement initiatives (11, 22, 23). Educational interventions are therefore particularly relevant when embedded within broader quality-improvement programs and locally operationalized care pathways (22).

In middle-income countries, particularly in Latin America, strengthening institutional capacity for early sepsis response is a high priority given the burden of disease and contextual implementation constraints. Nevertheless, regional evidence on feasible, formally evaluated educational and implementation strategies remains limited (2, 24). In our institution, the sepsis code has been implemented as part of a broader quality-improvement strategy, and its initial operational deployment in the emergency department was previously associated with lower in-hospital mortality in a pilot institutional evaluation (12). Within that broader institutional framework, the present study evaluated the effect of an educational implementation strategy on immediate sepsis code knowledge among hospital personnel in a high-complexity hospital in Colombia, using an expert-reviewed institutional knowledge instrument and complementary participant-level, item-level, and sensitivity analyses. We hypothesized that the educational strategy would be associated with a substantial immediate improvement in operational Code Sepsis knowledge among trained hospital personnel.

## Materials and methods

### Study design and setting

We conducted a single-group quasi-experimental before-and-after study to evaluate changes in institutional Code Sepsis knowledge after a standardized educational implementation strategy. The study was carried out between April and December 2025 at a high-complexity tertiary hospital in Bogotá, Colombia. Reporting was structured in accordance with principles applicable to observational and pre–post intervention studies, with emphasis on transparent specification of the intervention, analytical sample, outcome construction, and sensitivity analyses.

### Participants and educational intervention

The target population comprised clinical and non-clinical hospital personnel who voluntarily attended institutional Code Sepsis training sessions. The intervention consisted of a standardized 45-minute in-person multidisciplinary educational session delivered by the institutional sepsis team, including an epidemiologist and an intensivist. The session addressed the operational components of the institutional Code Sepsis strategy, including recognition and activation, blood-culture collection, lactate interpretation and follow-up, timely antimicrobial therapy, hemodynamic stabilization targets, source control, screening for carbapenemase-producing organisms, and coordination with the Antimicrobial Stewardship Program.

Participants completed an electronic knowledge assessment immediately before and immediately after the educational session using Microsoft Forms under the institutional license. All attendees with at least one assessment were retained for descriptive database characterization. The primary analytical sample was restricted to individuals with complete, strictly matched preintervention and postintervention assessments.

### Instrument development and content validity

Knowledge was assessed using a 12-item instrument developed *de novo* by the institutional Code Sepsis team specifically for the institutional Code Sepsis strategy. The questionnaire was not adapted from a previously published or externally validated instrument. Item content was derived from a predefined domain matrix aligned with the operational structure of the protocol and designed to represent the knowledge required for its institutional implementation. The final instrument covered six domains: general recognition and activation, initial diagnostic actions, serum lactate and follow-up, initial treatment and hemodynamic stabilization, source control, and complementary institutional components of Code Sepsis. The expert-reviewed instrument is provided in Supplementary Table S1.

Content validity was evaluated by seven independent experts, who assessed item relevance, clarity, and representativeness/coherence. Each criterion was rated using a four-point ordinal scale. Ratings of 3 or 4 were classified as acceptable and recoded as agreement for the calculation of the content validity indices. For each criterion, the item-level content validity index (I-CVI) was calculated as the proportion of experts assigning an acceptable rating to the item. Scale-level content validity was summarized using both the average of the item-level indices (S-CVI/Ave) and the universal-agreement approach (S-CVI/UA), following established content-validity methodology (25, 26). Criterion-specific I-CVIs were reported separately to identify potential areas requiring refinement. Overall item adequacy was determined using the mean of the relevance, clarity, and representativeness I-CVIs. These procedures were intended to establish evidence of content relevance and representativeness for the institutional purpose of the instrument, rather than comprehensive psychometric validity.

Before institutional deployment, the questionnaire underwent a pilot administration in 14 participants to assess comprehension, response flow, and operational feasibility, leading to minor wording refinements without altering the conceptual structure of the instrument. The pilot was used for cognitive and operational refinement and was not intended to estimate reliability, dimensionality, or criterion validity.

### Outcome definition and score construction

Each item was recoded as correct or incorrect according to the institutional answer key. The original expert-reviewed 12-item instrument yielded a total knowledge score ranging from 0 to 12, with higher values indicating a greater number of correct responses. During analytical data verification, a postintervention response-format artifact was identified for the institutional implementation-status item, which affected the interpretability of its paired score transition. Accordingly, the primary analytical outcome was defined as an 11-item total knowledge score excluding that item, with a possible range from 0 to 11. The 12-item total score was retained for a dedicated sensitivity analysis to evaluate the consistency of the findings under the original scoring structure.

The primary within-participant outcome was the paired postintervention minus preintervention difference in the 11-item score. At the item level, the outcome was the binary correctness status for each evaluable item in the primary score.

### Record linkage and analytical samples

Preintervention and postintervention questionnaires were linked through a strict matching procedure based on participant identifiers available in both forms. The principal analytical dataset included 591 strictly matched pre–post pairs. To evaluate robustness to the matching strategy, a prespecified high-confidence sensitivity dataset was generated by adding a limited set of auditable additional pairs identified through conservative identity and temporal criteria, yielding 609 paired observations. This dataset was used only for sensitivity analysis and not for the primary estimate.

Because inclusion in the paired analytical sample was not complete among all preintervention respondents, representativeness was examined by comparing paired and unpaired participants using standardized mean differences for selected baseline characteristics and baseline score distributions. In addition, an inverse probability weighting sensitivity analysis was conducted to assess whether differential inclusion in the matched analytical sample materially altered the estimated mean score change.

### Statistical analysis

The analytical methods were selected according to the paired pre–post design, the distribution and measurement level of each outcome, and the dependence structure of repeated observations. The choice of methods was independent of the statistical software used and was based on established statistical principles for paired, binary, and longitudinally correlated data.

Descriptive statistics were used to summarize score distributions at both assessment moments. Continuous scores were reported using means with standard deviations and medians with interquartile ranges. Paired score differences were summarized using means, medians, dispersion measures, and bootstrap 95% confidence intervals for both mean and median differences to obtain interval estimates without relying on a normality assumption (27). Because paired score differences were not normally distributed, the primary inferential comparison used the Wilcoxon signed-rank test. Effect magnitude was quantified using the rank-biserial correlation as a paired non-parametric effect-size measure.

Three score-based analyses were reported in parallel: the primary analysis using the 11-item score in the 591 strictly paired participants; sensitivity analysis A using the 11-item score in the 609 high-confidence paired participants; and sensitivity analysis B using the original 12-item score in the 591 strictly paired participants.

For item-level evaluation, preintervention and postintervention proportions of correct responses were calculated for each of the 11 items included in the primary score. Absolute changes were expressed in percentage points. Paired binary transitions were assessed using McNemar's test, which is appropriate for paired dichotomous responses, with false discovery rate adjustment according to the Benjamini–Hochberg procedure to limit the expected proportion of false discoveries across multiple item-level comparisons (28).

To estimate the global change in the probability of a correct response while accounting for repeated item-level observations within participants, a generalized estimating equations model with binomial family, logit link, exchangeable working correlation structure, and robust standard errors was fitted (29). The model included postintervention status as the main exposure and item indicators to adjust for baseline differences in item difficulty. The postintervention effect was reported as an odds ratio with 95% confidence interval.

For the inverse probability weighting sensitivity analysis, the probability of inclusion in the strict paired sample was estimated from preintervention participant characteristics and baseline knowledge score using a regularized logistic model. Stabilized weights were then applied to estimate the weighted mean paired score change. The weighted estimate was compared with the unweighted primary estimate to assess robustness to potential analytical-sample selection using established weighting principles (30).

Statistical analyses were conducted using Python in a Google Colaboratory environment. Python and Google Colaboratory served exclusively as the computational environment for implementing the prespecified analytical strategy. Data management and numerical procedures were performed with pandas and NumPy, while inferential analyses were implemented using SciPy, statsmodels, and scikit-learn. The software environment did not determine the selection of statistical methods. The analytical code supporting the reported results is available from the corresponding author upon reasonable request, subject to institutional authorization.

### Ethical considerations

The study protocol was reviewed and approved by the Corporate Research Ethics Committee of Fundación Santa Fe de Bogotá, Colombia (approval number CCEI-17785-2025; approved on May 14, 2025). The study was conducted in accordance with the ethical principles of the Declaration of Helsinki and applicable Colombian regulations for minimal-risk health research.

## Results

The final 12-item instrument demonstrated strong expert-based content validity. Across the seven expert reviewers, item-level content validity indices were uniformly high for relevance and representativeness, while clarity indices ranged from 0.714 to 1.000. Mean item-level I-CVI values ranged from 0.857 to 1.000. Although four items had clarity I-CVIs of 0.714, their relevance and representativeness indices remained high, and all items met the predefined overall adequacy criterion based on the mean I-CVI. The global S-CVI/Ave was 0.960, and the global S-CVI/UA was 0.833, supporting the overall content validity of the instrument for assessing operational Code Sepsis knowledge in this institutional context (Table 1).

**Table 1:** Content validity evidence for the expert-reviewed 12-item Code Sepsis knowledge instrument.

Item	Domain	*n*	Item-level content validity index				Classification
			Rel.	Clar.	Rep.	Mean I-CVI	
1	Domain 1. General recognition and Code Sepsis activation	7	1.000	1.000	1.000	1.000	Adequate
2	Domain 1. General recognition and Code Sepsis activation	7	1.000	1.000	1.000	1.000	Adequate
3	Domain 2. Initial diagnostic actions	7	1.000	1.000	1.000	1.000	Adequate
4	Domain 2. Initial diagnostic actions	7	1.000	0.714	1.000	0.905	Adequate
5	Domain 3. Serum lactate and follow-up	7	1.000	0.857	1.000	0.952	Adequate
6	Domain 3. Serum lactate and follow-up	7	1.000	1.000	1.000	1.000	Adequate
7	Domain 4. Initial treatment and hemodynamic stabilization	7	1.000	1.000	1.000	1.000	Adequate
8	Domain 4. Initial treatment and hemodynamic stabilization	7	1.000	1.000	1.000	1.000	Adequate
9	Domain 4. Initial treatment and hemodynamic stabilization	7	1.000	0.714	1.000	0.905	Adequate
10	Domain 5. Source control	7	1.000	1.000	1.000	1.000	Adequate
11	Domain 6. Complementary institutional components of Code Sepsis	7	0.857	0.714	1.000	0.857	Adequate
12	Domain 6. Complementary institutional components of Code Sepsis	7	1.000	0.714	1.000	0.905	Adequate

Criterion-specific and mean item-level content validity indices based on ratings from seven independent experts. Rel., relevance; Clar., clarity; Rep., representativeness/coherence; I-CVI, item-level content validity index; S-CVI, scale-level content validity index. All items were evaluated by seven experts. Global indices were S-CVI/Ave = 0.960 and S-CVI/UA = 0.833.

A total of 986 preintervention and 958 postintervention questionnaires were available. Strict record linkage yielded 591 complete pre–post pairs, corresponding to 59.9% of preintervention assessments and 61.7% of postintervention assessments. The principal analytical sample therefore comprised 591 participants with complete paired measurements. In the audit of sample representativeness, baseline preintervention total knowledge scores were similar between paired and unpaired respondents, with a standardized mean difference of 0.058. Some imbalances were observed in service composition and years of professional experience, particularly for pharmacy, hospital flow, nursing, and participants with more than 10 years of experience. These differences were documented to contextualize the paired analytical sample and were further addressed through inverse probability weighting sensitivity analysis (Supplementary Table S2).

In the primary analysis based on the 11-item score, participants showed a marked increase in knowledge after the institutional educational strategy. The mean preintervention score was 5.80 out of 11, compared with 9.22 after the session. The paired mean increase was 3.42 points, with a bootstrap 95% confidence interval from 3.20 to 3.64. This increase represented 31.1% of the total possible score range, providing a scale-based indication of the magnitude of the observed change. The median paired increase was 3.0 points, with an interquartile range from 2.0 to 5.0. The Wilcoxon signed-rank test indicated a statistically significant pre–post difference, and the rank-biserial correlation showed a very large positive paired effect, consistent with a dominant shift toward higher postintervention scores (Table 2).

**Table 2:** Pre–post change in the Code Sepsis knowledge score and robustness analyses.

Analysis	*n*	Mean difference (95% CI)	Median difference (95% CI)	IQR	Wilcoxon *W*	*p* value	Rank-biserial *r*
Primary: strict pairs, 11-item score	591	3.42 (3.20–3.64)	3.0 (3.0–3.0)	2.0–5.0	2,699.0	<0.001	0.9646
Sensitivity A: high-confidence pairs, 11-item score	609	3.40 (3.19–3.62)	3.0 (3.0–3.0)	1.0–5.0	2,795.5	<0.001	0.9654
Sensitivity B: strict pairs, 12-item score	591	3.18 (2.95–3.40)	3.0 (2.0–3.0)	1.0–5.0	4,132.0	<0.001	0.944

Primary analysis based on the strict paired analytical sample and prespecified sensitivity analyses. Differences were calculated as postintervention minus preintervention scores. Confidence intervals for mean and median differences were obtained by bootstrap resampling. IQR, interquartile range.

The direction-of-change analysis reinforced this pattern. Among the 591 strictly paired participants, 520 individuals improved their 11-item score, 39 remained unchanged, and 32 showed lower postintervention scores. This corresponded to improvement in 88.0% of participants, no change in 6.6%, and score reduction in 5.4%. Thus, participants who improved outnumbered those whose scores decreased by more than 16–1, showing that the favorable mean change was broadly distributed rather than concentrated in a small subgroup of high responders.

The score improvement remained highly consistent across sensitivity analyses. When the analytical sample was expanded to 609 high-confidence matched pairs, the mean paired increase in the 11-item score was 3.40 points, with a 95% confidence interval from 3.19 to 3.62. This estimate differed from the primary estimate by only 0.02 points. When the original 12-item instrument was analyzed in the 591 strict pairs, the mean paired increase was 3.18 points, with a 95% confidence interval from 2.95 to 3.40. Both sensitivity analyses produced large rank-biserial correlations and statistically significant Wilcoxon tests, closely mirroring the primary finding. The similarity of these estimates indicates that the main result was not meaningfully dependent on either the strict record-linkage rule or the exclusion of the implementation-status item from the primary score.

The inverse probability weighting analysis also supported the robustness of the primary estimate. The IPW-weighted mean paired increase in the 11-item score was 3.20 points, compared with 3.42 points in the unweighted primary analysis. The absolute difference between these estimates was 0.22 points, equivalent to approximately 6.4% of the unweighted mean change, indicating that the main conclusion was not materially altered after accounting for differential probability of inclusion in the matched sample (Supplementary Table S3). Although weighting modestly attenuated the estimated improvement, the weighted mean change remained substantial, and its 95% confidence interval remained entirely above zero.

All 11 items included in the primary score showed statistically significant improvement after false discovery rate correction. The largest absolute increase was observed for knowledge of Code Sepsis, which rose from 49.2% correct preintervention to 99.7% correct postintervention, an increase of 50.4 percentage points. Substantial gains were also observed for the mean arterial pressure target, which increased by 40.9 percentage points, time to source drainage, which increased by 39.6 percentage points, and blood volume per blood-culture bottle, which increased by 36.9 percentage points (Table 3). These were among the items with the greatest baseline knowledge deficits, and they showed the largest postintervention gains in highly actionable components of the protocol.

**Table 3:** Item-level changes in correct responses after the institutional Code Sepsis training strategy.

Item	Domain	Component	Correct responses			Discordant paired transitions		FDR-adjusted *p* value
			Pre, %	Post, %	Δ, pp	I → C, *n*	C → I, *n*	
1	General recognition and activation	Knowledge of Code Sepsis	49.2	99.7	50.4	299	1	<0.001
3	Initial diagnostic actions	Minimum number of blood-culture bottles	51.1	77.3	26.2	193	38	<0.001
4	Initial diagnostic actions	Blood volume per bottle	43.3	80.2	36.9	246	28	<0.001
5	Serum lactate and follow-up	Lactate reading and interpretation	60.9	93.1	32.1	198	8	<0.001
6	Serum lactate and follow-up	Follow-up lactate monitoring	31.8	62.3	30.5	225	45	<0.001
7	Initial treatment and stabilization	Mean arterial pressure target	32.3	73.3	40.9	256	14	<0.001
8	Initial treatment and stabilization	Timely antibiotic initiation	72.9	95.8	22.8	141	6	<0.001
9	Initial treatment and stabilization	Specific antibiotic sequencing	65.3	85.8	20.5	145	24	<0.001
10	Source control	Time to source drainage	24.5	64.1	39.6	269	35	<0.001
11	Complementary institutional components	Screening for carbapenemase carriers	73.9	92.9	19.0	126	14	<0.001
12	Complementary institutional components	ASP–Code Sepsis integration	74.5	97.5	23.0	142	6	<0.001

Paired item-level analysis in the strict analytical sample of 591 participants. Pre, preintervention; Post, postintervention; Δ, absolute change; pp, percentage points; I → C, incorrect-to-correct transition; C → I, correct-to-incorrect transition. *P* values were obtained using McNemar's test and adjusted using the Benjamini–Hochberg false discovery rate procedure.

Additional large increases were observed for lactate reading and interpretation, follow-up lactate monitoring, and the minimum number of blood-culture bottles, with absolute gains of 32.1, 30.5, and 26.2 percentage points, respectively. Items with comparatively higher baseline performance also improved meaningfully, including ASP–Code Sepsis integration, timely antibiotic initiation, specific antibiotic sequencing, and carbapenemase-carrier screening, with postintervention absolute gains ranging from 19.0 to 23.0 percentage points. Across all items, the number of participants transitioning from incorrect to correct substantially exceeded the number transitioning from correct to incorrect. The improvement across all six content domains indicates that the observed effect was not restricted to general awareness of Code Sepsis but extended to diagnostic, therapeutic, hemodynamic, microbiological, and source-control components.

**Figure 1:**
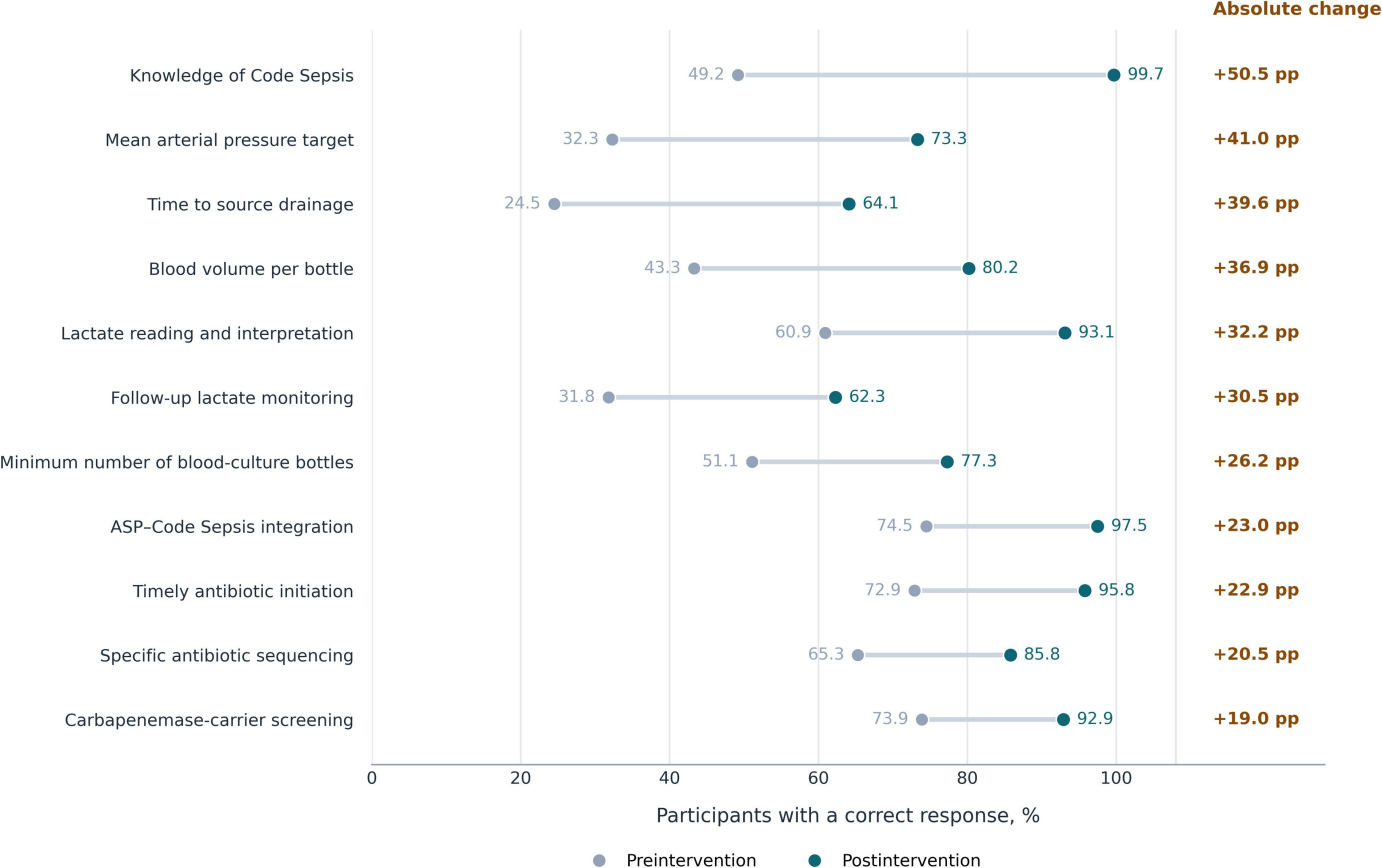
Item-level improvement in correct responses after the institutional Code Sepsis educational strategy. The figure displays the percentage of participants with a correct response before and after the intervention for each of the 11 items included in the primary analytical score. Items are ordered by absolute postintervention improvement. Values on the right indicate the absolute change in percentage points. ASP, antimicrobial stewardship program; pp, percentage points.

Figure 1 visually summarizes the magnitude and ordering of these gains, showing a consistent rightward shift in the proportion of correct responses after the educational strategy across every evaluated knowledge domain.

The generalized estimating equations model confirmed a strong overall postintervention increase in the odds of providing a correct response after accounting for item-level differences in baseline difficulty and within-participant correlation. Postintervention assessments were associated with 5.63-fold higher odds of a correct item-level response compared with preintervention assessments, with a 95% confidence interval from 5.05 to 6.28 and a *p* value below 0.001.

At the observed proportion level, correct item-level responses increased from 52.7% before the educational session to 83.8% after the session. This model-based result was consistent with the paired score analysis and with the item-specific McNemar findings, supporting a broad and coherent improvement in operational Code Sepsis knowledge after the institutional training strategy.

## Discussion

This study found that a brief institutional educational implementation strategy was associated with a substantial improvement in immediate sepsis code knowledge among hospital personnel in a high-complexity hospital. Using an expert-reviewed institutional knowledge instrument, the primary paired analysis showed a mean increase of 3.42 points in the 11-item analytical score, equivalent to 31.1% of the total possible score range, with 88.0% of participants improving their postintervention score. Only 5.4% obtained a lower score, indicating that the favorable change was broadly distributed across participants rather than being driven by a small subgroup. This pattern was reinforced by consistent gains across all evaluated items, a very large rank-biserial correlation, and a generalized estimating equations model showing markedly higher odds of a correct item-level response after training. Taken together, these findings indicate a favorable and operationally meaningful immediate gain in protocol knowledge, while not establishing sustained retention, behavioral change, or clinical benefit. These findings are consistent with the view that effective implementation of sepsis bundles depends not only on the availability of clinical recommendations, but also on organizational capabilities, interprofessional coordination, and workforce preparedness at the point of care (23).

A notable contribution of this study is that the educational effect was not confined to a single knowledge domain. Improvements were observed in general Code Sepsis recognition, diagnostic actions, lactate-related monitoring, hemodynamic stabilization targets, source control, and complementary institutional components. The largest item-level gains were observed in recognition of Code Sepsis, knowledge of the mean arterial pressure target, time to source drainage, and blood volume requirements for blood-culture bottles. These findings suggest that brief institutional training can improve both broad conceptual awareness and highly operational knowledge required for time-sensitive protocol execution.

The magnitude of improvement was substantial and operationally meaningful at the knowledge level. In the primary analysis, the mean score increased from 5.80 before the educational strategy to 9.22 after the session, with a paired mean difference of 3.42 points on an 11-item scale. This difference represented 31.1% of the full score range and was accompanied by improvement in 88.0% of participants, whereas only 5.4% obtained a lower postintervention score. The distribution of individual changes therefore suggests that the overall increase reflected a broad shift in the trained workforce rather than an average driven by a limited number of participants. In addition, the proportion of correct item-level responses increased from 52.7% to 83.8%, and postintervention responses were associated with 5.63-fold higher odds of correctness in the GEE model. The agreement between the participant-level score analysis and the item-level GEE model is important because the two approaches evaluate the intervention-associated change from complementary perspectives: the former summarizes overall knowledge acquisition within each participant, whereas the latter estimates the global change in response correctness while accounting for repeated items and differences in item difficulty.

The sensitivity analyses further support the stability of the finding. Expanding the linkage strategy changed the estimated mean improvement by only 0.02 points, while restoring the excluded implementation-status item yielded a slightly smaller but still substantial increase. Similarly, inverse probability weighting reduced the mean difference from 3.42 to 3.20 points, indicating modest attenuation after accounting for measured differences in paired-sample inclusion but no substantive change in interpretation. Collectively, these analyses reduce concern that the main finding arose primarily from the record-linkage procedure, the selected scoring structure, or measured selection into the paired analytical sample.

At the item level, the largest improvements occurred in areas with direct operational relevance, including Code Sepsis recognition, mean arterial pressure targets, timing of source control, blood-culture volume, and lactate monitoring. This pattern suggests that the session addressed both general protocol awareness and concrete actions required during time-sensitive sepsis care. Nevertheless, residual postintervention errors in some items identify potential targets for reinforcement, audit, and periodic retraining.

While clinical outcomes were not assessed, the literature suggests that educational efforts within sepsis initiatives can enhance care processes (22, 31). The ABISS-Edusepsis study documented improvements in empirical antibiotic treatment after a structured educational intervention, while a later multifaceted educational initiative improved anti-infectious care measures, reinforcing the role of knowledge strengthening as a prerequisite for optimizing sepsis-related clinical processes (31, 32). Accordingly, the present findings should be interpreted as evidence of successful immediate knowledge acquisition within an implementation strategy, not as direct evidence of improved bedside performance or patient outcomes.

From an implementation perspective, the study provides evidence for a low-burden, 45-minute educational strategy with institutional reach and immediate evaluation, aligning with approaches to close the gap between guidelines and practice through feasible, reproducible, and scalable interventions. Implementation determinants show that the success of strategies like the sepsis code depends on internal factors (culture, leadership, resources, workflows) and team integration of the innovation (33). The present findings complement this perspective by showing that an educational strategy embedded within an institutional Code Sepsis framework can measurably improve the protocol knowledge expected to support coordinated implementation.

The present findings should be interpreted within the context of a broader institutional sepsis quality-improvement strategy. In our hospital, the operational implementation of the sepsis code in the emergency department was previously associated with lower in-hospital mortality compared with a historical cohort (12). Within that broader framework, strengthening protocol knowledge among multidisciplinary personnel should not be viewed as an isolated educational objective, but rather as a plausible implementation mechanism to enhance protocol readiness, interprofessional coordination, and consistency of early response across hospital services.

These findings are particularly relevant in middle-income settings, where institutions often face the dual challenge of improving guideline implementation while adapting interventions to local workforce structures and operational constraints (34). Regional evidence regarding formally evaluated educational strategies for sepsis implementation remains limited (24). In that context, the present study contributes empirical data from a Latin American high-complexity hospital and supports the role of standardized training as a feasible component of institutional preparedness for early sepsis response.

Overall, these findings support the role of brief, standardized, multidisciplinary educational strategies as feasible components of institutional efforts to strengthen sepsis code readiness in high-complexity hospitals.

This study has several limitations. First, the quasi-experimental before-and-after design without a concurrent control group limits causal inference and precludes ruling out temporal or contextual influences during the implementation period. Second, the outcome was immediate post-intervention knowledge; therefore, the findings cannot be extrapolated to longer-term knowledge retention, behavioral change, adherence to sepsis bundles, or patient-level outcomes. Third, the immediate re-administration of the same instrument may have introduced testing effects, and participant awareness of being evaluated may have contributed to a Hawthorne effect (35). Finally, although the instrument underwent expert review and pilot testing, it was developed as a multidomain operational measure for local implementation purposes rather than as a formal psychometric scale; accordingly, its interpretation should be limited to the assessment of immediate protocol knowledge in this specific context. In addition, the institutional implementation-status item was excluded from the primary score because of a postintervention response-format artifact; however, the consistency of the 12-item sensitivity analysis supports the robustness of the main inference.

## Conclusion

In this high-complexity hospital in Colombia, a brief, standardized, multidisciplinary educational implementation strategy was associated with a marked immediate improvement in operational Code Sepsis knowledge among hospital personnel. The consistency of the findings across paired score analyses, item-level comparisons, generalized estimating equations, and sensitivity analyses supports the robustness of this association. These results support the integration of scalable educational components into institutional sepsis quality-improvement strategies aimed at strengthening workforce readiness for early recognition and coordinated response.

## Data Availability

The raw data supporting the conclusions of this article will be made available by the authors, without undue reservation.

## References

[1] Singer M Deutschman CS Seymour CW Shankar-Hari M Annane D Bauer M. The third international consensus definitions for sepsis and septic shock (sepsis-3). J Am Med Assoc. (2016) 315:801. 10.1001/jama.2016.0287 PMC4968574 26903338

[2] Rudd KE Johnson SC Agesa KM Shackelford KA Tsoi D Kievlan DR. Global, regional, and national sepsis incidence and mortality, 1990–2017: analysis for the global burden of disease study. Lancet. (2020) 395:200–11. 10.1016/S0140-6736(19)32989-7 31954465 PMC6970225

[3] Liu V Escobar GJ Greene JD Soule J Whippy A Angus DC. Hospital deaths in patients with sepsis from 2 independent cohorts. J Am Med Assoc. (2014) 312:90–2. 10.1001/JAMA.2014.5804 24838355

[4] Rodríguez F Barrera L De La Rosa G Dennis R Dueñas C Granados M. The epidemiology of sepsis in Colombia: a prospective multicenter cohort study in ten university hospitals. Crit Care Med. (2011) 39:1675–82. 10.1097/CCM.0B013E318218A35E 21685740

[5] Jaramillo GD Ramirez SM. USER protocol as a guide to resuscitation of the patient with septic shock in the emergency department. Open Access Emerg Med. (2021) 13:33–43. 10.2147/OAEM.S289148 33603505 PMC7886247

[6] Reinhart K Daniels R Kissoon N Machado FR Schachter RD Finfer S. Recognizing sepsis as a global health priority—a WHO resolution. N Engl J Med. (2017) 377:414–7. 10.1056/NEJMP1707170 28658587

[7] Evans L Rhodes A Alhazzani W Antonelli M Coopersmith CM French C. Surviving sepsis campaign: international guidelines for management of sepsis and septic shock 2021. Intensive Care Med. (2021) 47:1181–247. 10.1007/S00134-021-06506-Y 34599691 PMC8486643

[8] Weiss SL Peters MJ Oczkowski SJW Belley-Cote E Buysse C Choong KLM. Surviving sepsis campaign international guidelines for the management of sepsis and septic shock in children 2026. Intensive Care Med. (2026) 52:937–83. 10.1007/s00134-026-08360-2 41870559

[9] Levy MM Evans LE Rhodes A. The surviving sepsis campaign bundle: 2018 update. Intensive Care Med. (2018) 44:925–8. 10.1007/s00134-018-5085-0 29675566

[10] Levy MM Rhodes A Phillips GS Townsend SR Schorr CA Beale R. Surviving sepsis campaign: association between performance metrics and outcomes in a 7.5-year study. Intensive Care Med. (2014) 40:1623–33. 10.1007/s00134-014-3496-0 25270221

[11] Damiani E Donati A Serafini G Rinaldi L Adrario E Pelaia P. Effect of performance improvement programs on compliance with sepsis bundles and mortality: a systematic review and meta-analysis of observational studies. PLoS One. (2015) 10:e0125827. 10.1371/JOURNAL.PONE.0125827 25946168 PMC4422717

[12] Jaramillo GD Morales LMC Useche CAV. Implementation of a sepsis code protocol at an academic institution in Colombia: a pilot study. J Clin Med. (2026) 15:767. 10.3390/JCM15020767 41598704 PMC12842312

[13] Seymour CW Gesten F Prescott HC Friedrich ME Iwashyna TJ Phillips GS. Time to treatment and mortality during mandated emergency care for sepsis. N Engl J Med. (2017) 376:2235–44. 10.1056/NEJMOA1703058 28528569 PMC5538258

[14] Palencia Herrejón E González Del Castillo J Ramasco Rueda F Candel FJ Sánchez Artola B von Wernitz Teleki A. Documento de consenso para la implantación y desarrollo del Código Sepsis en la Comunidad de Madrid. Rev Esp Quimioter. (2019) 32:400–9. PMID: 31345006 31345006 PMC6719654

[15] Yébenes JC Lorencio C Esteban E Espinosa L Badia JM Capdevila JA. Código Sepsis Interhospitalario en Catalunya: modelo organizativo territorial para la atención inicial al paciente con sepsis. Med Intensiva. (2020) 44:36–45. 10.1016/j.medin.2019.05.008 31542182

[16] Carbajo RS Palma J Martin-Loeches I. The next frontier in sepsis: connected ICU data for real-world clinical decision making. Intensive Care Med. (2026) 52:301–8. 10.1007/S00134-025-08284-3 41586891 PMC12971731

[17] Ferreras Amez JM Arribas Entrala B Sarrat Torres MA García Noaín A Caudevilla Martínez A Colás Orós C. Evaluación de los resultados antes y después de la implantación del Código Sepsis en Aragón. Emergencias. (2017) 29:154–60. PMID: 28825234 28825234

[18] Carlbom DJ Rubenfeld GD. Barriers to implementing protocol-based sepsis resuscitation in the emergency department–results of a national survey. Crit Care Med. (2007) 35:2525–32. 10.1097/01.CCM.0000298122.49245.D7 18075366

[19] Poeze M Ramsay G Gerlach H Rubulotta F Levy M. An international sepsis survey: a study of doctors’ knowledge and perception about sepsis. Crit Care. (2004) 8:R409–13. 10.1186/CC2959 15566585 PMC1065059

[20] Assunção M Akamine N Cardoso GS Mello PVC Teles JMM Nunes ALB. Survey on physicians’ knowledge of sepsis: do they recognize it promptly? J Crit Care. (2010) 25:545–52. 10.1016/J.JCRC.2010.03.012 20646902

[21] Roye-Green K Willis R Priestley SR Vickers I. Knowledge, practice and attitudes to the management of sepsis in Jamaica. J Crit Care Med. (2022) 8:232–41. 10.2478/JCCM-2022-0024 PMC9682927 36474617

[22] Choy CL Liaw SY Goh EL See KC Chua WL. Impact of sepsis education for healthcare professionals and students on learning and patient outcomes: a systematic review. J Hosp Infect. (2022) 122:84–95. 10.1016/J.JHIN.2022.01.004 35045340

[23] Ferrer R Artigas A Levy MM Blanco J González-Díaz G Garnacho-Montero J. Improvement in process of care and outcome after a multicenter severe sepsis educational program in Spain. J Am Med Assoc. (2008) 299:2294–303. 10.1001/JAMA.299.19.2294 18492971

[24] Jaimes F. The declaration of São Paulo: sepsis as the main cause of preventable death and disability in Latin America. A call to action in order to reduce the burden of sepsis. Colomb J Anesthesiol. (2019) 47:76–7. 10.1097/CJ9.0000000000000090

[25] Polit DF Beck CT Owen SV. Is the CVI an acceptable indicator of content validity? Appraisal and recommendations. Res Nurs Health. (2007) 30(4):459–67. 10.1002/nur.20199 17654487

[26] Lynn MR. Determination and quantification of content validity. Nurs Res. (1986) 35:382–6. 10.1097/00006199-198611000-00017 3640358

[27] Johnson RW. An introduction to the bootstrap. Teach Stat. (2001) 23:49–54. 10.1111/1467-9639.00050

[28] Benjamini Y Hochberg Y. Controlling the false discovery rate: a practical and powerful approach to multiple testing. J R Stat Soc Series B Stat Methodol. (1995) 57:289–300. 10.1111/j.2517-6161.1995.tb02031.x

[29] Liang KY Zeger SL. Longitudinal data analysis using generalized linear models. Biometrika. (1986) 73:13–22. 10.1093/biomet/73.1.13

[30] Austin PC Stuart EA. Moving towards best practice when using inverse probability of treatment weighting (IPTW) using the propensity score to estimate causal treatment effects in observational studies. Stat Med. (2015) 34:3661–79. 10.1002/sim.6607 26238958 PMC4626409

[31] Ferrer R Martínez ML Gomà G Suárez D Álvarez-Rocha L De La Torre MV. Improved empirical antibiotic treatment of sepsis after an educational intervention: the ABISS-edusepsis study. Crit Care. (2018) 22:167. 10.1186/S13054-018-2091-0 29933756 PMC6013897

[32] Schwarzkopf D Matthaeus-Kraemer CT Thomas-Rüddel DO Rüddel H Poidinger B Bach F. A multifaceted educational intervention improved anti-infectious measures but had no effect on mortality in patients with severe sepsis. Sci Rep. (2022) 12:3925. 10.1038/S41598-022-07915-9 35273276 PMC8913650

[33] Damschroder LJ Reardon CM Widerquist MAO Lowery J. The updated consolidated framework for implementation research based on user feedback. Implement Sci. (2022) 17:75. 10.1186/S13012-022-01245-0 36309746 PMC9617234

[34] Rudd KE Kissoon N Limmathurotsakul D Bory S Mutahunga B Seymour CW. The global burden of sepsis: barriers and potential solutions. Crit Care. (2018) 22:232. 10.1186/s13054-018-2157-z 30243300 PMC6151187

[35] McCambridge J Witton J Elbourne DR. Systematic review of the Hawthorne effect: new concepts are needed to study research participation effects. J Clin Epidemiol. (2014) 67:267–77. 10.1016/J.JCLINEPI.2013.08.015 24275499 PMC3969247

