# Supplementary material for: A standardized educational implementation strategy improves operational Code Sepsis knowledge in a high-complexity hospital in Colombia
Source: Front Health Serv. 2026 Aug 4;6:1907012. doi: 10.3389/frhs.2026.1907012 (PMC13481772; doi:10.3389/frhs.2026.1907012)
Supplement: Supplementary file 1 [file Table1.docx]

| **Supplementary Table S1. Final expert-reviewed Code Sepsis knowledge instrument** | | | | |
| --- | --- | --- | --- | --- |
| Twelve-item instrument, including domains, final wording, response formats, and institutional answer keys. | | | | |
| Item | Domain | Final item wording | Response format | Institutional answer key |
| 1 | Domain 1. General recognition and Code Sepsis activation | Do you know what Code Sepsis is? | Closed categorical response | Yes |
| 2 | Domain 1. General recognition and Code Sepsis activation | Does Code Sepsis exist in your institution? | Closed categorical response | Under implementation, according to the institutional configuration at the time of application |
| 3 | Domain 2. Initial diagnostic actions | According to the institutional Code Sepsis protocol, how many blood-culture bottles should be collected? | Single-best-answer selection | 4 bottles |
| 4 | Domain 2. Initial diagnostic actions | According to the institutional Code Sepsis protocol, what is the minimum blood volume required in each blood-culture bottle? | Single-best-answer selection | 10 mL per bottle |
| 5 | Domain 3. Serum lactate and follow-up | According to the institutional Code Sepsis protocol, within what time frame should serum lactate be read and interpreted? | Single-best-answer selection | 1 hour |
| 6 | Domain 3. Serum lactate and follow-up | According to the institutional Code Sepsis protocol, within what time frame should serum lactate be monitored after the initial approach? | Single-best-answer selection | 6 hours |
| 7 | Domain 4. Initial treatment and hemodynamic stabilization | What is the minimum mean arterial pressure target during the initial stabilization of a patient with sepsis? | Single-best-answer selection | 65 mmHg |
| 8 | Domain 4. Initial treatment and hemodynamic stabilization | According to the institutional Code Sepsis protocol, what is the recommended time frame for antibiotic initiation in a patient with suspected sepsis? | Single-best-answer selection | 1 hour |
| 9 | Domain 4. Initial treatment and hemodynamic stabilization | Is it important to administer antibiotics in a specific sequence during the initial Code Sepsis approach? | Closed categorical response | Yes |
| 10 | Domain 5. Source control | According to the institutional Code Sepsis protocol, within what time frame should a drainable infectious source be controlled? | Single-best-answer selection | 6 hours |
| 11 | Domain 6. Complementary institutional components of Code Sepsis | Does your institution perform screening for patients carrying carbapenemase-producing organisms? | Closed categorical response | Yes |
| 12 | Domain 6. Complementary institutional components of Code Sepsis | Do Antimicrobial Stewardship Programs integrate with the institutional Code Sepsis strategy? | Closed categorical response | Yes |
| **Note.** The 12-item instrument underwent expert-based content validation before empirical application. For the primary analytical score, the institutional implementation-status item was excluded because of a response-format artifact in the postintervention database; however, it remains part of the validated instrument. | | | | |

| **Supplementary Table S2. Representativeness audit of the strict paired analytical sample** | | | | | | |
| --- | --- | --- | --- | --- | --- | --- |
| Comparison of selected baseline characteristics between paired and unpaired preintervention participants. | | | | | | |
| Characteristic | Category / metric | Paired sample | Unpaired sample | Absolute difference, pp | Absolute SMD | SMD flag |
| Clinical/operational service | Nursing | 307 (51.9) | 241 (61.0) | 9.1 | 0.184 | 0.10–0.19 |
| Clinical/operational service | Pharmacy | 67 (11.3) | 17 (4.3) | 7.0 | 0.264 | ≥0.20 |
| Clinical/operational service | Hospital flow | 49 (8.3) | 9 (2.3) | 6.0 | 0.271 | ≥0.20 |
| Clinical/operational service | Laboratory | 9 (1.5) | 14 (3.5) | 2.0 | 0.129 | 0.10–0.19 |
| Clinical/operational service | Medical | 96 (16.2) | 65 (16.5) | 0.2 | 0.006 | <0.10 |
| Clinical/operational service | Other | 3 (0.5) | 8 (2.0) | 1.5 | 0.136 | 0.10–0.19 |
| Clinical/operational service | Residents/students | 16 (2.7) | 4 (1.0) | 1.7 | 0.126 | 0.10–0.19 |
| Clinical/operational service | Missing | 2 (0.3) | 28 (7.1) | 6.8 | 0.363 | ≥0.20 |
| Clinical/operational service | Respiratory therapy | 42 (7.1) | 9 (2.3) | 4.8 | 0.23 | ≥0.20 |
| Years of professional experience | 1–2 years | 77 (13.0) | 24 (6.1) | 7.0 | 0.238 | ≥0.20 |
| Years of professional experience | 3–5 years | 124 (21.0) | 62 (15.7) | 5.3 | 0.137 | 0.10–0.19 |
| Years of professional experience | 6–7 years | 39 (6.6) | 22 (5.6) | 1.0 | 0.043 | <0.10 |
| Years of professional experience | 8–10 years | 83 (14.0) | 66 (16.7) | 2.7 | 0.074 | <0.10 |
| Years of professional experience | >10 years | 131 (22.2) | 119 (30.1) | 8.0 | 0.182 | 0.10–0.19 |
| Years of professional experience | Missing | 137 (23.2) | 102 (25.8) | 2.6 | 0.061 | <0.10 |
| Baseline total knowledge score | 12-item score before analytical exclusion of the implementation-status item | n=591; mean 6.08 (SD 2.74); median 6.0 [IQR 4.0–8.0] | n=395; mean 6.25 (SD 3.00); median 7.0 [IQR 5.0–9.0] |  | 0.058 | <0.10 |
| **Note.** Standardized mean differences were used descriptively to assess the magnitude of imbalance between paired and unpaired participants. The baseline total knowledge score was comparable between groups before paired analysis. pp = percentage points; SD = standard deviation; IQR = interquartile range; SMD = standardized mean difference. | | | | | | |

| **Supplementary Table S3. Sensitivity analysis addressing paired-sample inclusion using inverse probability weighting** | | | | |
| --- | --- | --- | --- | --- |
| Comparison between the primary unweighted estimate and the IPW-weighted estimate of pre–post score change. | | | | |
| Analysis | Participants, n | Mean paired difference (95% CI) | Median paired difference (95% CI) | IQR |
| Primary unweighted analysis | 591 | 3.42 (3.20 to 3.64) | 3.0 (3.0 to 3.0) | 2.0 to 5.0 |
| IPW-weighted sensitivity analysis | 591 | 3.20 (2.99 to 3.41) | 3.0 (3.0 to 3.0) | 2.0 to 5.0 |
| **Note.** Inverse probability weights were derived from the regularized inclusion model. The IPW-weighted estimate remained closely aligned with the primary unweighted estimate, supporting the robustness of the main paired analysis. IPW = inverse probability weighting; IQR = interquartile range. | | | | |
